# Experimental glioma with high bHLH expression harbor increased replicative stress and are sensitive toward ATR inhibition

**DOI:** 10.1093/noajnl/vdaa115

**Published:** 2020-09-10

**Authors:** Marilin Sophia Koch, Stefan Czemmel, Felix Lennartz, Sarah Beyeler, Srinath Rajaraman, Justyna Magdalena Przystal, Parameswari Govindarajan, Denis Canjuga, Manfred Neumann, Patrizia Rizzu, Stefan Zwirner, Michael Stefan Hoetker, Lars Zender, Bianca Walter, Marcos Tatagiba, Olivier Raineteau, Peter Heutink, Sven Nahnsen, Ghazaleh Tabatabai

**Affiliations:** 1 Department of Neurology and Interdisciplinary Neuro-Oncology, Hertie Institute for Clinical Brain Research, University Hospital Tübingen, Eberhard Karls University Tübingen, Tübingen, Germany; 2 Quantitative Biology Center (QBiC), Eberhard Karls University Tübingen, Tübingen, Germany; 3 German Center for Neurodegenerative Diseases (DZNE), German Center for Neurodegenerative Diseases, Tübingen, Germany; 4 Department of Internal Medicine VIII, University Hospital Tübingen, Eberhard Karls University Tübingen, Tübingen, Germany; 5 Department of Internal Medicine I, University Hospital Tübingen, Eberhard Karls University Tübingen, Tübingen, Germany; 6 German Translational Cancer Consortium (DKTK), DKFZ partner site Tübingen, Tübingen, Germany; 7 Department of Neurosurgery, University Hospital Tübingen, Eberhard Karls University Tübingen, Tübingen, Germany; 8 University of Lyon, Université Claude Bernard Lyon 1, Inserm, Stem Cell and Brain Research Institute U1208, Bron, France

**Keywords:** bHLH transcription factors, CAGE, DDR, E47, RNA-Seq

## Abstract

**Background:**

The overexpression of (basic)helix-loop-helix ((b)HLH) transcription factors (TFs) is frequent in malignant glioma. We investigated molecular effects upon disruption of the (b)HLH network by a dominant-negative variant of the E47 protein (dnE47). Our goal was to identify novel molecular subgroup-specific therapeutic strategies.

**Methods:**

Glioma cell lines LN229, LNZ308, and GS-2/GS-9 were lentivirally transduced. Functional characterization included immunocytochemistry, immunoblots, cytotoxic, and clonogenic survival assays in vitro, and latency until neurological symptoms in vivo. Results of cap analysis gene expression and RNA-sequencing were further validated by immunoblot, flow cytometry, and functional assays in vitro.

**Results:**

The induction of dnE47-RFP led to cytoplasmic sequestration of (b)HLH TFs and antiglioma activity in vitro and in vivo. Downstream molecular events, ie, alterations in transcription start site usage and in the transcriptome revealed enrichment of cancer-relevant pathways, particularly of the DNA damage response (DDR) pathway. Pharmacologic validation of this result using ataxia telangiectasia and Rad3 related (ATR) inhibition led to a significantly enhanced early and late apoptotic effect compared with temozolomide alone.

**Conclusions:**

Gliomas overexpressing (b)HLH TFs are sensitive toward inhibition of the ATR kinase. The combination of ATR inhibition plus temozolomide or radiation therapy in this molecular subgroup are warranted.

**Key Points**

- Disruption of (b)HLH transcription factor networks affects DDR signaling.
- Gliomas overexpressing (b)HLH transcription factors are sensitive to ATR inhibition.

**Importance of the Study**

The heterogeneous genomic landscape of glioblastoma leads to therapeutic challenges. We targeted (b)HLH transcription factors, ie, a complete molecular network in experimental glioma and identified the DNA damage signaling as a therapeutically relevant druggable pathway in the subgroup of bHLH-overexpressing glioma.

Glioblastoma remains a cancer with limited registered therapeutic options. Current multimodal therapies include surgical resection and radiochemotherapy leading to a median overall survival in the range of 1.5 years.^1^ Attempts to improve the overall survival by adding antiangiogenic drugs like Cilengitide,^2^ Bevacizumab,^3^ or EGFRvIII-targeting strategies^4^ to the therapeutic armory have not improved overall survival. Thus, novel paradigms to identify biologically relevant therapeutic strategies are urgently needed.

In this study, we used our model system^5^ that is based on the disruption of a key molecular network in glioblastoma, ie, the transcriptional network of (basic)helix-loop-helix (ie, (b)HLH) transcription factors (TFs) by mutated E proteins. We aimed at exploring downstream effects of (b)HLH transcriptional network disruption to identify vulnerabilities and potential druggable targets. Helix-loop-helix (HLH) and basic helix-loop-helix (bHLH) TF proteins are involved in a variety of cellular processes.^6^ They also represent key mediators in the pathophysiology of several malignancies^7^ including glioblastoma.^8,9^ For example, bHLH TFs play a key role responsible for maintaining a reprogramming machinery. Their common structural features include a DNA-binding domain, the necessity to form homo- or heterodimers^6^ and E-box sites within promoter regions to modulate transcription of specific target genes.^10^

E47 is a member of the bHLH family,^6^ an alternative splicing product of E2A^6,11^ and plays a crucial role in cancer,^12^ neural development, and neural differentiation.^13–15^ The overexpression of a dominant-negative E47 (dnE47), lacking its nuclear translocation signal leads to apoptosis, reduction of growth-capacity in vitro, and prolongation of symptom-free survival in vivo.^5^

Here, we investigated downstream molecular events upon dnE47-mediated cytoplasmic bHLH sequestration by a comprehensive promoterome and transcriptome analysis. Our hypothesis is that this approach might help to discover novel therapeutic approaches in glioblastoma.

## Materials and Methods

### Cell Lines

Long-term cell lines (LN229 and LNZ308) were cultured with Dulbecco’s modified Eagle medium (DMEM) 10% fetal calf serum (FCS) and 0.1% Gentamicin (ThermoFisher) (complete DMEM). Glioma stem like cells (GS-2 and GS-9)^16^ were cultured in Neurobasal Medium (ThermoFisher), 2% B27 (ThermoFisher), 20 ng/mL recombinant human epidermal growth factor and fibroblast growth factor (Peprotech), 1% l-Glutamine (Sigma), and 0.1% Gentamicin (ThermoFisher).

### Lentivirus Production

The generation of the dnE47-RFP and E47-RFP vector was previously described.^17,18^ For virus production, 1.5 × 10^7^ 293T cells per flask were seeded on day 0. On day 1, a mixture of expression, envelope and packing plasmid together with polyethylenimine (Sigma) was added and incubated at 37°C, 5% CO_2_ for 4 h, medium was removed, exchanged with complete DMEM. Virus-containing supernatants were harvested and ultra-centrifuged, and 10–200 MOI were used for transduction of cells.

### Immunoblots

The generation of separate cytoplasmic and nuclear protein fractions were as follows: For the induction of cell lysis and extraction of the cytoplasmic fraction, cells were suspended in buffer A (containing 10 mM 4-(2-hydroxyethyl)-1-piperazineethanesulfonic acid [HEPES] pH 7.7, 10 mM KCl, 0.1 mM ethylenediaminetetraacetic acid [EDTA], 0.1 mM ethylene glycol-bis(2-aminoethylether)-N,N,N′,N′-tetraacetic acid [EGTA], 1 mM dithiothreitol [DTT], and 0.5 mM phenylmethylsulfonyl fluoride [PMSF]); afterwards the remaining cell pellet was suspended in buffer C (containing 20 mM HEPES pH 7.7, 0.4 mM KCl, 1 mM EDTA, 1 mM EGTA, 1 mM DTT, and 1 mM PMSF) for disruption of the nucleic membrane and isolation of the nuclear fraction. Protein concentration was determined by performing Bradford’s assay. Immunoblot was performed with subcellular fractions at timepoints 0 and 24, 48 and 72 h after Dox-mediated induction. For depiction of protein expression of RFP (1:1000, MBL) and ID1(1:1000, Biocheck) in nucleus and cytoplasm, the corresponding antibodies were used. IkBα and β-Tubulin served as loading controls.

Further antibodies included pATR (S428, 1:1000, Cell Signaling), pATM (Ser1987, 1:1000, Invitrogen), pCHK1 (S317 1:1000, Cell Signaling), pCHK2 (T383, 1:1000, Abcam), pRPA2 (1:1000, Abcam), and RFPA2 (1:1000, Abcam). Tubulin served as loading control. Analysis was carried out by Bio-Rad imager (Bio-Rad).

All gels were imaged with Bio-Rad imager (Bio-Rad).

### Immunocytochemistry

Cells were stained with Phalloidin-fluorescein isothiocyanate (1:2, Sigma) and 4′,6-diamidino-2-phenylindole (1:20 000, ThermoFisher) and mounted with Mowiol (Vector). Primary antibody was anti-ID1 (1:500, rabbit monoclonal anti-mouse/human ID1, Biocheck, resp. rabbit polyclonal anti-mouse/human ID1, Abcam), the secondary species-matched antibody was conjugated to AlexaFluor 647 (1:500, anti-rabbit IgG Invitrogen). Images were acquired with confocal microscopy (Zeiss confocal LSM 510).

### Cytotoxicity Assay

Cells were seeded at 5000 cells/well in triplicates. All therapies were performed with the indicated concentrations. Cells were treated for 72 h and incubated with CellTiter Blue (1:6, Promega) at 37°C. The fluorescent signal was measured with a fluorometer (GloMax explorer, Promega). Statistical analysis—1-way analysis of variance (ANOVA) with Dunnett’s multiple comparisons test—was performed with GraphPad Prism (version 5/7).

### Clonogenicity Assays

For monotherapy, 24 h after seeding either temozolomide (0–2 µM) or radiation (0–2 Gy) or doxycycline (1:500) was administered in serum-free medium. After 48 h, a medium change with serum-free medium with doxycycline was performed. After 72 h, medium was again changed to FCS-containing medium with or without doxycycline.

For combination therapy DOX-RT/CT, 24 h after seeding doxycycline was administered (1:500) under serum-free conditions. After 48 h cells were treated with either temozolomide (0–2 µM) or radiation (0–2 Gy) or the combination in serum-free medium. The day after a medium change into serum-containing medium with doxycycline was performed.

For combination therapy RT/CT-DOX, 24 h after seeding, cells were treated with either temozolomide (0–2 µM) or radiation (0–2 Gy) or the combination the in serum-free medium. After 48 h cells were treated with doxycycline (1:500) in serum-free medium. The day after a medium change into serum-containing medium with doxycycline was performed.

After 10 days, cells were fixed and stained with a Crystal violet containing solution (phosphate-buffered saline [PBS] 1×, 1% formaldehyde, 0.5% Crystal violet, and 10% methanol). Colony area was determined according to previous publications.^19^ Statistical analysis—1-way ANOVA with Dunnett’s multiple comparisons test—was performed with GraphPad Prism (version 5/7).

### Animal Models

All animal procedures were approved by the facility for animal protection, veterinary service, and lab animal biology of the Eberhard-Karls-University Tübingen and performed in accordance with German law. GS-2 cells (150 000 cells in 3 µl) were stereotactically implanted in the right striatum of female athymic mice (CD1nu/nu). On day 15, mice were randomized into 2 groups. Doxycycline (2 mg/mL) or 1% sucrose were applied through drinking water. Endpoints included a weight loss of 15% or the development of reduced spontaneous-explorative behavior.

### RNA-Sequencing (RNA-Seq)

RFP-dnE47 transduced LN229 cells were treated with doxycycline and harvested after 24, 48, and 72 h. RNA extraction was performed with RNeasy Kit (Qiagen) according to the manufacturer’s protocol. Sequencing was performed by the company CeGaT (https://www.cegat.de) in Tübingen, Germany using 100 ng of quality controlled RNA (RIN value = 10 for all 48 samples) to prepare libraries with the Illumina TruSeq stranded mRNA Library Prep Kit. The libraries were then sequenced on a HiSeq4000 machine in a 2 × 100 bp paired end mode.

### Cap Analysis Gene Expression (CAGE)

For CAGE the same RNA samples as for RNA-Seq were used. Sequencing was performed at the “Deutsches Zentrum für Neurodegenerative Erkrankungen (DZNE)” in Tübingen, Germany. CAGE libraries were prepared using a previously published protocol.^20^ Briefly, total RNA from the 48 samples was used as starting material and sequenced on a HiSeq2500 instrument in single end mode with 50 bp of read length.

CAGE reads were demultiplexed and trimmed using the adapter trimmer software Skewer.^21^ Then, CAGE reads were filtered for artifacts using TagDust.^22^ There was no filtering step for RNA-Seq samples. Afterwards the CAGE and RNA-Seq reads were mapped to the human genome (hg38), both using the STAR aligner.^23^ Using the STAR alignment bam files, raw reads were counted on genes using featureCounts together with a GTF file downloaded from Ensembl (version 38.94). Analysis downstream of the mapping step for CAGE and RNA-Seq was mainly performed in R (version 3.4.3). For CAGE data, the “CAGEr” package (version 1.20)^24^ was used to group the mapped reads into CAGE defined transcriptional start sites (CTSS). As the data looked well distributed, CAGE raw data were not further normalized to keep raw tags for downstream statistical analysis. Using the CAGEr package, these raw values from individual CTSS were clustered into tag clusters (TCs) by a simple distance-based clustering method joining CTSS sites if they are closer than 20 bp to each other. Afterwards, consensus promoters/clusters were created across samples from the TCs.

For downstream statistical analysis, the R package “Limma” (version 3.34.9) was used. For CAGE and RNA-Seq, the raw read table with counts for 54 794 genes and the raw tag count table for 129 608 consensus clusters, respectively, were used as input for “Limma.” Next for CAGE, the consensus cluster counts for the same gene were summed up using the R package “doBy” and its function summaryBy(). Then, for RNA-Seq and CAGE these input lists were filtered and genes with constant 0 counts were removed leaving 38 659 genes for RNA-Seq and 29 478 genes for CAGE. Then for both, CAGE and RNA-Seq, the main experimental factors genotype (with levels dnE47 and RFP), treatment (with levels plus Dox and minus Dox), and time (with levels 0, 24, 48, and 72 h) were combined into a single factor which allowed to extract all the comparisons of interest including the 4 interaction terms: diff_0h = (dnE47_plusDox_0h-RFP_plusDox_0h) − (dnE47_minusDox_0h-RFP_minusDox_0h), diff_24h = (dnE47_plusDox_24h-RFP_plusDox_24h) − (dnE47_ minusDox_24h-RFP_minusDox_24h), diff_48h = (dnE47_ plusDox_48h-RFP_plusDox_48h) − (dnE47_minusDox_48h- RFP_minusDox_48h), and diff_72h = (dnE47_plusDox_ 72h-RFP_plusDox_72h) − (dnE47_minusDox_72h-RFP_ minusDox_72h). These interaction/difference terms allowed to answer the question whether a gene responds differently to treatment in the 2 genotypes at each given timepoint.

Statistics, as detailed above, were produced for all genes. From this list, potential best candidates were chosen that show a Benjamini–Hochberg corrected *P* adjusted value <.05 in any of the 4 interaction terms described above.

Using this list of differentially expressed (DE) genes for both datasets as input, the “biomaRt” package in R was used to map them to unique Entrez IDs. These Entrez IDs were then used for kyoto encyclopedia of genes and genomes (KEGG) pathway enrichment analysis which was performed in R using the function enrichKEGG() from the package “clusterProfiler.” Pathways were defined to be significantly enriched when the False Discovery rate (qvalue) for each pathway did not exceed 5%. Analysis was conducted with differentially expressed genes for each time-dependent expression pattern (eg, with genes only differentially expressed at 24 h but not at any other timepoint) or combined into a single list of unique gene IDs. Pathway maps were created using the function pathview() from the R package “pathview” by plotting the KEGG graphs and color the DE genes in each pathway according to the direction of their log fold changes in each of the 4 interaction/difference terms described with green (negative fold change for the interaction term) and red (positive fold change for the interaction term).

Following statistics, annotation was performed on all consensus clusters from the CAGE dataset using the CHIPeakAnno package (version 3.12.7) in order to obtain the distance to the nearest transcriptional start site (TSS) for each cluster. As annotation data, a GTF file was used downloaded from Ensembl (version 38.84). Heatmaps and other plots were all done in R mainly using the R packages gplots (version 3.0.1) and ggplot2 (version 3.1.0).

Promoter shifting analysis has been done using the R package CAGEr with pairwise comparisons between 2 groups of samples, namely dnE47 + Dox versus RFP + Dox and dnE47-Dox versus RFP-Dox at each of the 4 timepoints. For a functional enrichment analysis focused on TF binding, genes with a significant promoter shift >0.15 were analyzed with the g:Profiler webtool (version e98_eg45_p14_ce5b097, 12/25/2019).

### Flow Cytometry

LN229 cells seeded in triplicates were treated with either AZD6738 (1.2 µM), temozolomide (87 µM), combination, or mock for 72 h in the indicated concentrations. Harvested cells were stained with Annexin V (Pacific blue, Invitrogen) and propidium iodide (PI). Flow cytometry was performed with MACSQuant (Miltenyi Biotec), data were analyzed with FlowJo (FlowJo LLC, Version 10).

### Cell Cycle Analysis

LN229 seeded in duplicates were treated with either AZD6738 (1.2 µM), temozolomide (87 µM), combination, or mock for 24 h in the indicated concentrations. Harvested cells were incubated with PI staining solution (50 µg/mL PI, 0.2% Triton X-100, 100 µg RNase A, PBS, 1 g/l glucose) for 15 min. Flow cytometry was performed with MACSQuant (Miltenyi Biotec), data were analyzed with FlowJo (FlowJo LLC, Version 10).

### Statistical Analysis

If not stated otherwise, statistical analysis was performed with GraphPad Prism (version 5/7). Synergy calculation was performed as previously described.^25^

## Results

### Mutated E Protein Led to Cytoplasmic Sequestration of ID1 In Vitro

**Figure 1.**
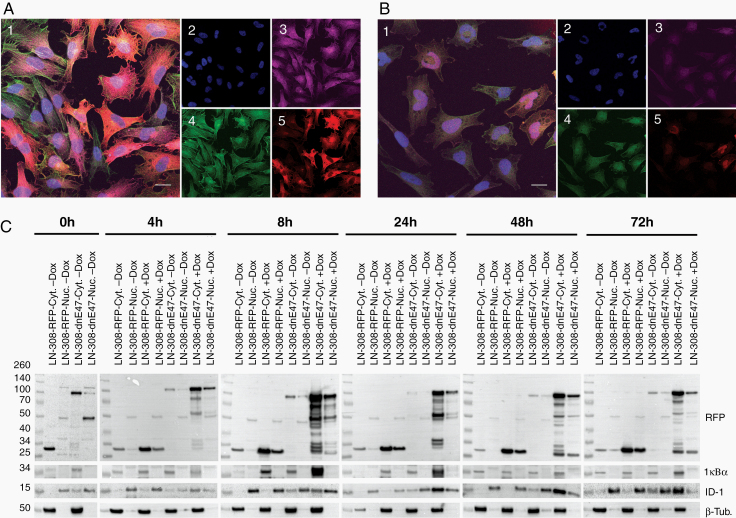
Cytoplasmic sequestration of bHLH TF. (A) Immunocytochemistry of LN229 dnE47-RFP and (B) LN229 E47-RFP. Confocal microphotograph, merged microphotographs (1) and 4′,6-diamidino-2-phenylindole (2), ID1 (3), and Phalloidin (4). RFP distribution in cells (5) (bar = 20 µm). (C) Increased cytoplasmic sequestration of ID1. Immunoblots with nuclear (Nuc.) and cytoplasmatic (Cyt.) protein fractions of LNZ308 RFP-dnE47 and RFP-E47; IkBα (cyt) and β-Tubulin (Nuc) as internal controls.

LN229, LNZ308, and GS-2 glioma cells were lentivirally transduced with RFP-conjugated dnE47 or respective RFP-conjugated wildtype (wt) E47. Upon doxycycline induction, the nuclear staining of ID1 decreased. Conversely, cytoplasmatic signal increased (Figure 1A and B) and LNZ308 (data not shown) after 48 h. We also observed this cytoplasmic sequestration after 48 h in GS-2 cells (Supplementary Figure S1). We then used fractionated lysates for immunoblots, separately investigating the cytoplasmic (Cyt) and nuclear (Nuc) compartments (Figure 1C) at 0–72 h. We observed a gradual increase in the cytoplasmic fraction of ID1 and RFP confirming efficient dnE47-mediated cytoplasmic sequestration over time (Figure 1C).

### Mutated E Protein Reduced Clonogenic Survival, Cellular Viability In Vitro, and Prolonged the Latency Until the Onset of Neurologic Symptoms In Vivo

**Figure 2.**
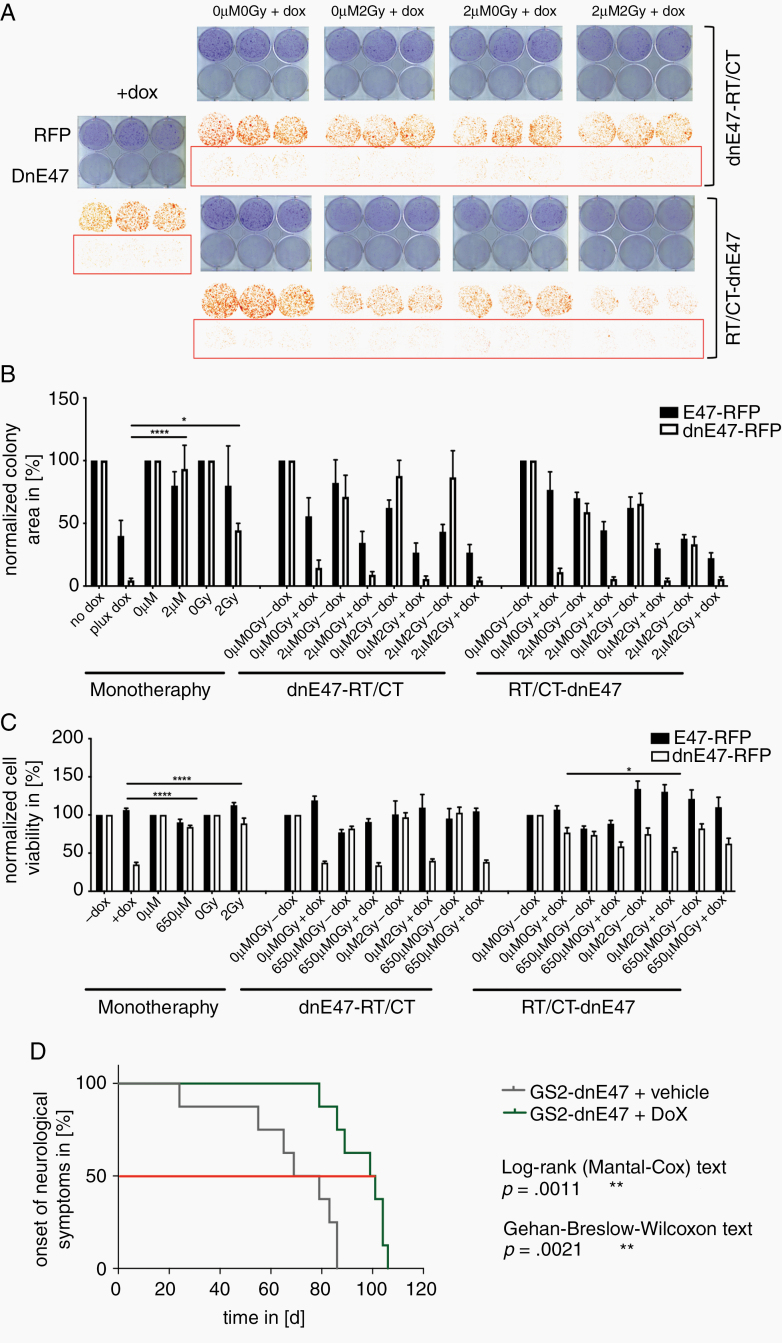
The antiglioma activity of mutated E protein. (A) Representative clonogenic survival plates and (B) quantifications of clonogenic survival of RFP-dnE47 and RFP-E47 transduced LN229 cells after indicated treatments. (C) Cytotoxicity assay of RFP-dnE47 and RFP-E47 transduced LNZ308 cells after indicated treatments. As dnE47 induction alone already strongly curtailed cell survival (dnE47 vs. RT *P* = .0384*, dnE47 vs. CT *P* < .0001****) resp. increased cytotoxic cell death (dnE47 vs. RT *P* < .0001****, dnE47 vs. CT *P* < .0001****) significantly better than chemo- or radiotherapy, neither preceding (RT-CT/DOX) nor subsequent (DOX/RT-CT) radiochemotherapy led to a consistent significant additional benefit. (D) Kaplan -Meier curves.

Next, we performed clonogenicity and cytotoxicity assays after dnE47 induction alone or in combination with conventional therapies, ie, irradiation and temozolomide (Figure 2). Yet, as dnE47 induction alone already strongly curtailed cell survival by ~60–90%, neither preceding (RT-CT/DOX) nor subsequent (DOX/RT-CT) radiochemotherapy led to an additional benefit (Figure 2A–C). Further, in a GS-2 xenograft mouse model, dnE47 expression significantly prolonged the latency until the onset of neurological symptoms (Figure 2D).

### Mutated E Protein Induced Time-Dependent Promoter Shifts

To further understand how dnE47 expression conveys its effect upon bHLH TF sequestration, we performed CAGE and RNA-Seq of LN229 glioma cells at different timepoints of dnE47 expression. Using the CAGE sequencing data, it was possible to infer differential usage of TSSs also known as “promoter shifting,” which may indicate changes in the regulation of transcription from the respective promoter.

In total, 2001 consensus clusters with a shifting score ≥0.15 were detected indicating that TSSs within these consensus clusters/promoter regions were used differently. Across all timepoints except 0 h, more shifting promoters were detected in dnE47-RFP versus E47-RFP + Dox in contrast to dnE47-RFP versus E47-RFP-Dox indicating increased alternative promoter usage of genes upon Dox treatment, suggesting a time-dependent dnE47-effect.

**Figure 3.**
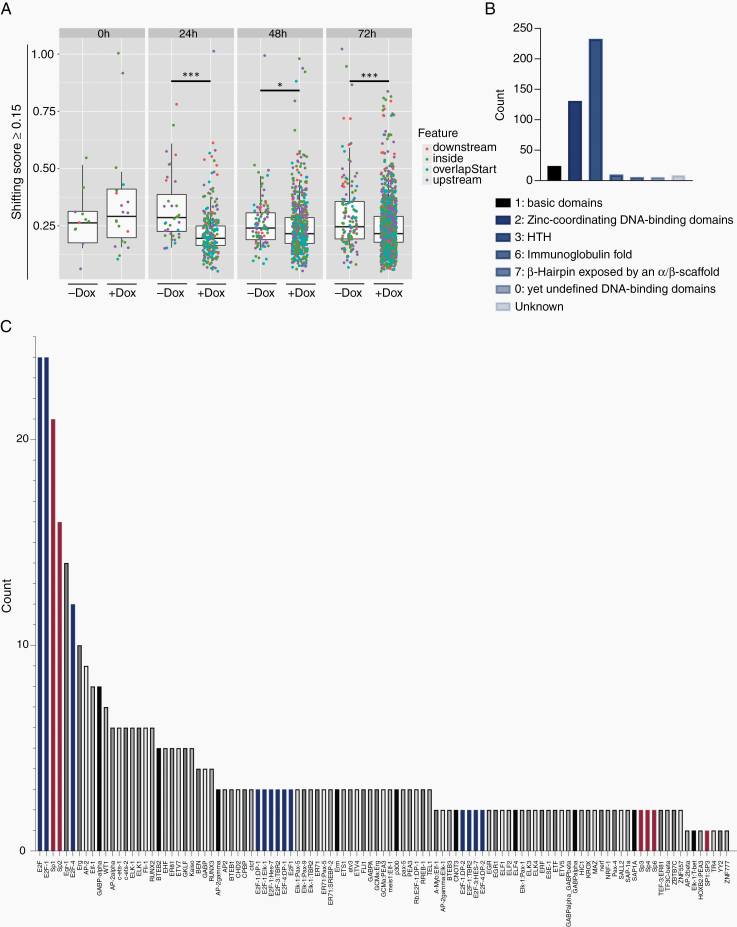
CAGE analysis. (A) Promoter shifting analysis. Boxplots show consensus clusters with a shifting score ≥0.15. Significant increase of shifting promoters at timepoints 24, 48, and 72 h after Dox induction. Statistical testing was performed with the Mann–Whitney test: 24 h (*P* value: <.0001 (***)), 48 h (*P* value: .03 (*)), and 72 h (*P* value: .0004 (***)). Shifting promoters were associated with transcriptionally relevant genome annotation features “overlap start” (blue), “inside” (green), “downstream” (orange), “upstream” (violet). *X*-axis labels: +Dox = dnE47-RFP + Dox versus E47-RFP + Dox; −Dox = dnE47-RFP-Dox versus E47-RFP-Dox. (B and C) TF-binding enrichment analysis after 72 h of those consensus clusters with a shifting score ≥0.15 showed a time-related increase in TF activity (B), especially for class 1 (basic domains), class 2 (Zinc-coordinating DNA-binding domains), and class 3 (helix-turn-helix domains). An enrichment of specific TF-binding domains was observed for the E2F (C, blue bars) and Sp-family (C, red bars).

By analyzing the mean values of the shifting scores, we observed a lower shifting score after doxycycline treatment compared with the control samples. Statistical testing using Mann–Whitney confirmed this finding and showed a significant difference in mean values for the +Dox and −Dox comparisons for timepoints 24 h (*P* value: <.0001 (***)), 48 h (*P* value: .03 (*)), and 72 h (*P* value: .0004 (***)) (Figure 3A).

These shifting promoters were mostly associated with transcriptionally relevant genome annotation features such as “overlap start” (TSS peak overlaps with the start of the gene) or “upstream” (TSS peak resides upstream of the gene) but also “inside” (TSS peak resides inside the gene) (see color legend in Figure 3A).

A TF-binding enrichment analysis showed a time-dependent accumulation of general TF activity with an increase in binding of helix-turn-helix domains (class 3 TF), Zinc-coordinating DNA-binding domains (class 2 TF) and basic domains (class 1) (Figure 3B).

Surrounding the shifted TSSs at 72 h, we observed enrichment for specific TF-binding motifs, mostly for the E2F and Sp-family. Clustering TF-binding motifs by family over all timepoints, we found increased counts for AP-2 (class 1 TF), BTEB2, Egr-1, Sp1, Sp2, Sp3, WT1, ZF5 (class 2 TF), E2F-1, E2F-3, E2F-4, ETF (class 3 TF), and BEN (class 0 TF) (Figure 3C).

### Mutated E Protein Induces Transcriptional Changes in Glioma Cells and Reveals New Druggable Targets

**Figure 4.**
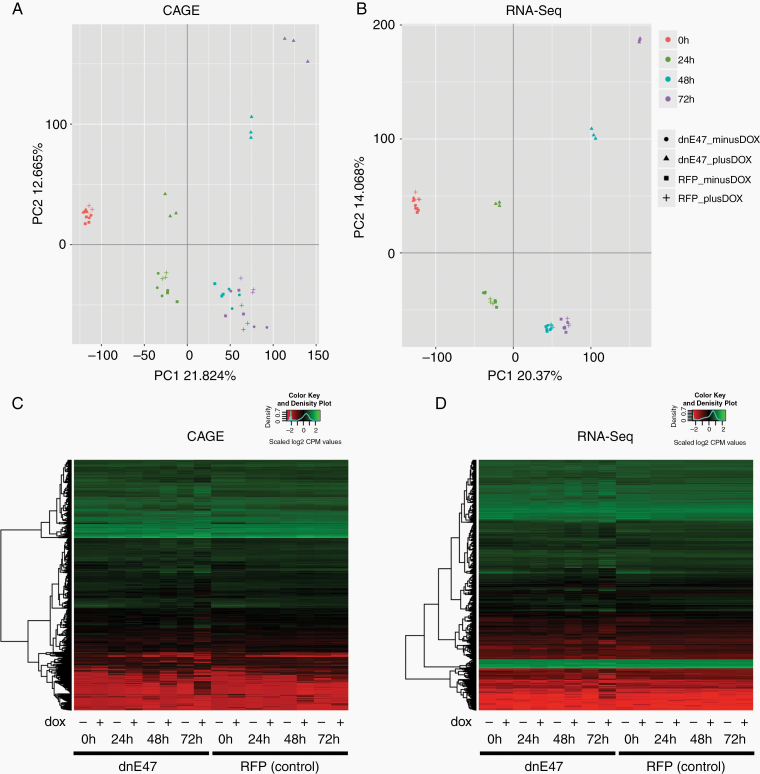
Integration of CAGE and RNA-Seq data. PCA of CAGE (A) and RNA-Seq data (B). PCA on CAGE (A) and RNA-Seq (B) filtered and normalized (log2 counts per million (CPM)) gene expression values in all 48 samples. The percentage of variation explained by PC1 and PC2 is indicated at each axis. Colors indicate samples from different timepoints and shapes from different genotype and treatment combinations. RFP-dnE47 samples sequester from the other samples after addition of doxycycline both in CAGE (A) and RNA-Seq (B) through course of time, while the other control cell samples cluster together. (C) Heatmaps of the differential expressed genes found in CAGE data. (D) Heatmaps of the differential expressed genes found in RNA-Seq data. Heatmap of the changes in expression of the 10 690 DE genes from the CAGE and of the 14 891 DE genes from the RNA-Seq dataset. The dendrogram on the left in (C and D) illustrates the clustering using euclidean distance. No clustering based on columns (samples) was performed. The distribution of the normalized expression values is shown as color key on the top right. Note that these values were also scaled on rows to have mean 0 and standard deviation 1.

We further complemented our CAGE data by RNA-sequencing. We then integrated our CAGE and RNA-Seq data. The principal component analysis (PCA) of RNA-Seq and CAGE data showed a very similar pattern with a segregation of dnE47-RFP protein samples after dnE47 activation with doxycycline over the course of time (Figure 4A and B). Interestingly, E47-RFP control samples as well as the dnE47 samples without doxycycline treatment started to cluster together after 24 h of doxycycline induction, whereas doxycycline treated dnE47 samples continued to segregate. The variance captured in PCA analysis for both RNA-Seq and CAGE was similar with ~34%, demonstrating a highly concordant influence of the experimental factors genotype (mutated or wildtype form of E47), time and treatment (with or w/o DOX) on gene expression profiles measured either by a technology based on capped 5′ ends (CAGE) or random fragments of RNA molecules (RNA-Seq). It can therefore be speculated that the observed sample clustering is indeed mainly influenced by a time-dependent induction of dnE47.

Normalized read counts were yielded for 38 659 unique gene IDs for RNA-Seq and 29 478 gene IDs for CAGE. Irrespective of time, in total 10 690 genes were differentially expressed in CAGE and 14 891 in RNA-Seq with a common set of 6910 which were differentially expressed in both methods when comparing dnE47 and RFP controls. The trend visible in the PCA analysis was also pronounced when visualizing differentially expressed genes using heatmaps (Figure 4C and D). The majority of the differentially expressed genes showed a clear shift in expression pattern not at 0 h but at all later timepoints after doxycycline induction (+ labeled samples) in RFP-dnE47 cells (left section of the heatmap) when compared with samples without doxycycline treatment (− labeled samples) and RFP-E47 control cells (right section of the heatmap) (Figure 4C and D).

The complete RNA-Seq and CAGE differential expression datasets—irrespective of their time-dependent expression pattern—were then used to identify pathway alterations mainly responsible for the dnE47-mediated effect by performing a KEGG pathway enrichment analysis. In total, 134 and 105 KEGG pathways were enriched for RNA-Seq and CAGE, respectively, and 92 KEGG pathways were significantly enriched in both RNA-Seq and CAGE. These pathways clustered into 11 different groups including cell fate, repair mechanisms, degradation related pathways, cancer pathways, signaling pathways, viral infection, bacterial infection, cell metabolism, cell structure, drug resistance, and other (Supplementary Figure S2A). In order to break down this list of pathways to those consistently enriched with time of doxycycline dependent dnE47 activation in both RNA-Seq and CAGE, a second time-dependent pathway analysis focused on those that showed an enrichment either based on genes differentially expressed only at 24 h (group I), at both 48 and 72 h (group II) or from 24 to 72 h (group III) after the beginning of doxycycline exposure (Supplementary Figure S2B).

**Figure 5.**
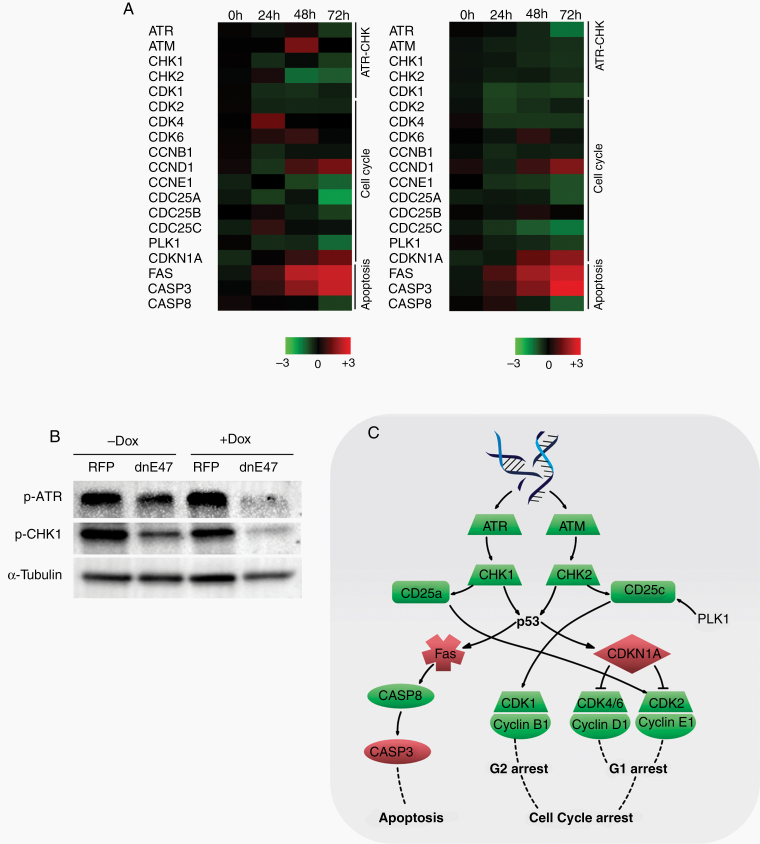
The DNA damage response pathway is one effector pathway. (A) Heatmap showing enriched genes for the DNA damage pathway. Color-coding is based on differential log fold change expression values (dnE47 vs. RFP) with red representing higher signal and green representing lower signal relative to the mean. (B) Immunoblot for ATR, CHK1, and CHK2. (C) Schematic overview of the ATR DNA damage pathway.

There were no significantly enriched pathways for CAGE or RNA-Seq in group I while group II consisted of 1 common pathway. As the 14 common pathways found significantly enriched in group III were based on genes which were consistently differentially expressed from 24 to 72 h, they were considered as most robust regarding validity of the dnE47-mediated effect on the transcriptome. Among these time-independent (Supplementary Figure S2A) and time-dependent (Supplementary Figure S2B) KEGG pathway enrichment analyses, we found alterations with highest significance for DNA replication, cell cycle, p53 signaling pathway, and apoptosis. Detailed analysis revealed key elements of cell cycle, apoptosis, and p53 signaling pathways—ataxia telangiectasia and Rad3 related (ATR) and CHK1 and their downstream effectors—to be differentially expressed (Figure 5A). Furthermore, we observed differential gene expressions for other members of the ATR–CHK1/DNA damage pathway, suggesting this pathway to be one of the main mediators of the dnE47-induced antiglioma effects.

### Mutated E Protein Leads to Altered ATR–CHK1 Signaling

Key elements of the ATR–CHK1 pathway (Figure 5C) were significantly downregulated particularly ATR (Figure 5A). We validated this finding by immunoblots, showing a similar effect on ATR–CHK1 pathway on the protein level (Figure 5B). Furthermore, pRpa2 (as an indicator of replicative stress) was reduced upon dnE47 induction (Supplementary Figure S3).

**Figure 6.**
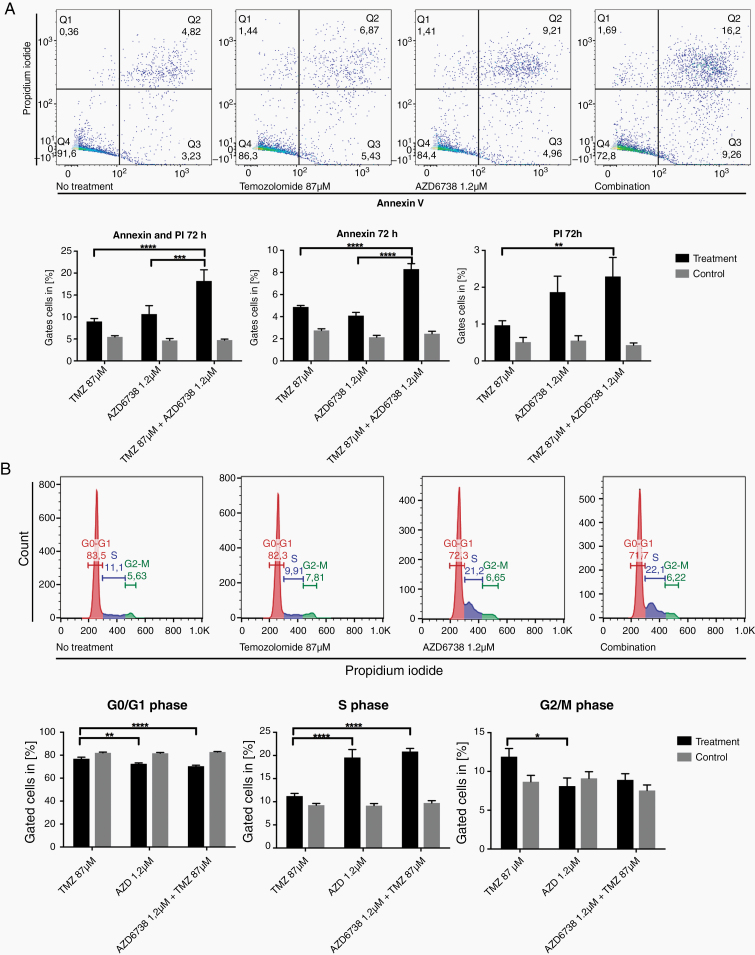
Pharmacological validation with an ATR inhibitor. (A) Flow cytometry with Annexin V/PI staining after the indicated treatments. Treatment with temozolomide alone leads to a modest increase of early (Q3) and late apoptotic cells (Q2), these fractions are higher in AZD6738 monotherapy. Combination of both therapeutics leads to a significant increase of early (Q3) and late apoptotic (Q2) cells compared with monotherapies. Statistical analysis with 2-way ANOVA and Tukey’s multiple comparisons test confirms these findings to be statistically significant (****P < .0001, ***P < .001, **P < .01). (B) Cell cycle analysis after treatment with AZD6738, temozolomide, or combinatorial treatment. Statistical analysis with 2-way ANOVA and Tukey’s multiple comparisons test confirms significant alterations in cell cycle distribution (****P < .0001, ***P < .001, **P < .01, *P < .05).

We performed a pharmacological validation with AZD6738 to inhibit ATR. Flow cytometry with Annexin V/PI showed enhanced apoptotic effects for the combination of ATR inhibition and temozolomide in Annexin V+ early (2-way ANOVA with Tukey’s multiple comparison’s test: *P* < .0001 and *P* < .0001 for comparison to temozolomide or ATR inhibition monotherapy) and Annexin V+/PI+ late (*P* < .0001 and *P* = .0008 for comparison to temozolomide or ATR inhibition monotherapy) apoptotic cells compared with monotherapies (Figure 6A). Furthermore, cell cycle flow cytometry showed a significant accumulation in S-phase after treatment with AZD6738 alone and in combination with temozolomide compared with temozolomide monotherapy (*P* < .0001 for temozolomide vs. AZD6738, *P* < .0001 for temozolomide vs. temozolomide + AZD6738). Conversely, a significant reduction of cells in G0/G1 phase was observed after treatment with AZD6738 and in combination with Temozolomide (*P* = .0039 for temozolomide vs. AZD6738 and *P* < .0001 for temozolomide vs. temozolomide + AZD6738) (Figure 6B).

## Discussion

Rationales for therapeutic strategies against malignant glioma are often identified by basic science knowledge about pathways regulating key malignant features, eg, angiogenesis or proliferation. The road to clinical translation includes the identification of druggable members of these molecular pathways. We used a different strategy and targeted a network, ie, the (b)HLH TFs and demonstrated antiglioma effects.^5^ Yet, pharmacological targeting of the whole (b)HLH transcriptional network in patients will not be feasible. In the present study, we therefore focused on the molecular mechanisms after the induction of a mutated E protein to identify biologically relevant and druggable targets.

We observed distinct transcriptomic alterations affecting multiple cancer related pathways (Figure 5; Supplementary Figure S2). In addition, CAGE data extended our view on the transcriptome in these cells and indicated that dnE47 induction is associated with a time-dependent differential usage of transcription start sites, which also showed an enrichment for TF-binding sites for the E2F and Sp-family TFs. Even though previous studies indicated that transcription start sites are frequently differentially used in cancer,^26,27^ the number of available CAGE datasets for cancer is still very limited. For example, in the current ENCODE release (v95, released at January 31, 2020) 78 CAGE datasets exist, but only 4 are from brain samples (the neuroblastoma cell line SK-N-SH), resulting in potentially lower accuracy of correlations between TF motif abundance and their actual frequency of usage.

Expression data from both CAGE and RNA-Seq demonstrate significant expression alterations of genes involved in cell cycle progression and DNA damage control after dnE47 activation. In dnE47-positive cells, we saw a significant downregulation of the ATR–CHK1 (ATR, ATM, CHK1, and CHK2) pathway and cell cycle checkpoint proteins (CDK1, CDK2, CDK4, CCNB1, and CCNE1). A connection between cell cycle regulation and E47 was previously proposed.^28^ An enforced expression of E47 in an E2A-deficient lymphoma cell line led to altered expression of cell cycle associated genes like CDK6, CDKN1A, and GADD45.^28^

Although relations between bHLH TF DEC1 and the DNA damage pathway have been shown (degradation of DEC1 controls the DNA damage response^29^), connections between E47, ID1, and ATR–CHK1 have not yet been exploited for therapeutic strategies in glioma. Previous studies suggested interactions of Olig2 with the DNA damage pathway: Olig2-mediated proliferation and glioma stem cell propagation is achieved by repression of CDKN1A,^30–32^ which is a downstream target of DNA damage induced p53 activation and regulates cell cycle progression.^33,34^ As high-throughput analyses after dnE47 activation showed significant alterations of the KEGG pathways cell cycle, Fanconi anemia and p53 pathway and their commonly shared ATR–CHK1 pathway, this builds a very promising basis for ATR inhibition as a potential therapeutic concept in glioma therapy. Immunoblot (Figure 5B) and Annexin V/PI flow cytometry (Figure 6) further validated this finding. Of note, proliferation is not altered upon dnE47 induction in glioma cells,^5^ and the main antiglioma effects are reduced clonogenic survival and increased apoptosis. Yet, the induction of dnE47 leads to reduced pRpa2 (Supplementary Figure S3), indicating higher replicative stress in bHLH-expressing cells underscoring the potential utility of ATR inhibition.

The effectors of the DNA damage pathway—ATR and ataxia telangiectasia mutated (ATM)—get activated by single and double strand breaks.^35^ Subsequent phosphorylation of downstream effectors like CHK1 and CHK2 eventuates in a cell cycle arrest at G1/S- and G2/M-phase^35^ (Figure 5C), enabling the cell to fix existing DNA damages before progressing the cell cycle. Conventional clinical treatment strategies in glioblastoma include temozolomide and radiation. They induce double strand breaks. Of note, recent data indicate that the ATR inhibitor AZD6738 is able to penetrate the blood brain barrier,^36^ and that ATR knockdown leads to significant sensitization to temozolomide in a glioma cell line.^37^ This approach has also been studied in other cancers: ATR inhibition with AZD6738 combined with other therapies proved to be synergistic for pancreatic cancer (ATRi and Gemcitabine),^38^ Her2-positive breast cancer (ATRi and Cisplatin),^39^ and non-small cell lung cancer (ATRi and Cisplatin).^40^ In radiotherapy of glioblastoma, replication stress leading to constitutive activation of the DNA damage response pathway under irradiation is thought to mediate radioresistance.^41,42^ Radioresistance in CD133-expressing glioma cells, is mediated via activation of the DNA damage pathway, making its inhibition a promising opportunity to circumvent this tumor escape mechanism.^43^ Yet, a combination of AZD6738 with radiation in vivo revealed no prolonged survival in this model.^36^

We conclude that the dnE47-mediated targeting of bHLH transcription network can serve as a model system to identify novel therapeutic approaches. Based on our data, we conclude that ATR inhibition will be a promising candidate particularly in glioma with high bHLH expression and in combination with treatments inducing double strands breaks. ATR inhibitors are in early stages of clinical development (NCT03188965). Based on our data, further clinical development steps of ATR inhibitors should include glioblastoma with high bHLH expression.

## Supplementary Material

vdaa115_suppl_Supplementary_Figures

## Notes

**Conflict of interest statement**. G.T. reports personal fees for advisory board participation and consulting from AbbVie, Bayer, Bristol-Myers-Squibb, Novocure, speakers fees from Medac, travel grants from Bristol-Myers-Squibb, Novocure, Medac; research grants from Roche Diagnostics and Medac. M.S.K. has received a scholarship from Medac and travel grants from Roche. The other authors report that they do not have any conflicts of interest to disclose.

## Notes

**Authorship Statement**. Study conception and design: G.T., M.S.K., S.B., S.C., and S.N. Acquisition of data: M.S.K., S.C., F.L., S.B., S.R., J.M.P., D.C., M.N., S.Z., and B.W. Analysis and interpretation of data: G.T., M.S.K., S.C., S.B., J.M.P., M.S.H., P.G., and S.R. Drafting of manuscript and revisions, approval of final version: all authors.
