# Supplementary material for: Experimental glioma with high bHLH expression harbor increased replicative stress and are sensitive toward ATR inhibition
Source: Neurooncol Adv. 2020 Sep 10;2(1):vdaa115. doi: 10.1093/noajnl/vdaa115 (PMC7592426; doi:10.1093/noajnl/vdaa115)

**Supplementary Figure S1: Immunocytochemistry of GS2 cells**

Immunocytochemistry of GS2 RFP-dnE47 (A) and GS2 RFP-E47 (B). Confocal microphotograph with Zeiss confocal LSM 510. Merged microphotographs are displayed in a composite (1). Staining was performed with DAPI (2), ID1 (3) and Phalloidin (4). All cells were transduced RFP (5). RFP-dnE47 transduced cells show a high fluorescent signal of RFP in cytoplasm with sparing of the nucleus in comparison to RFP-E47 transduced control cells in long-term and GS cell lines. In RFP-dnE47 GS2 cells, the fluorescent signal of ID1 spares the nucleus. Bar = 20µm.


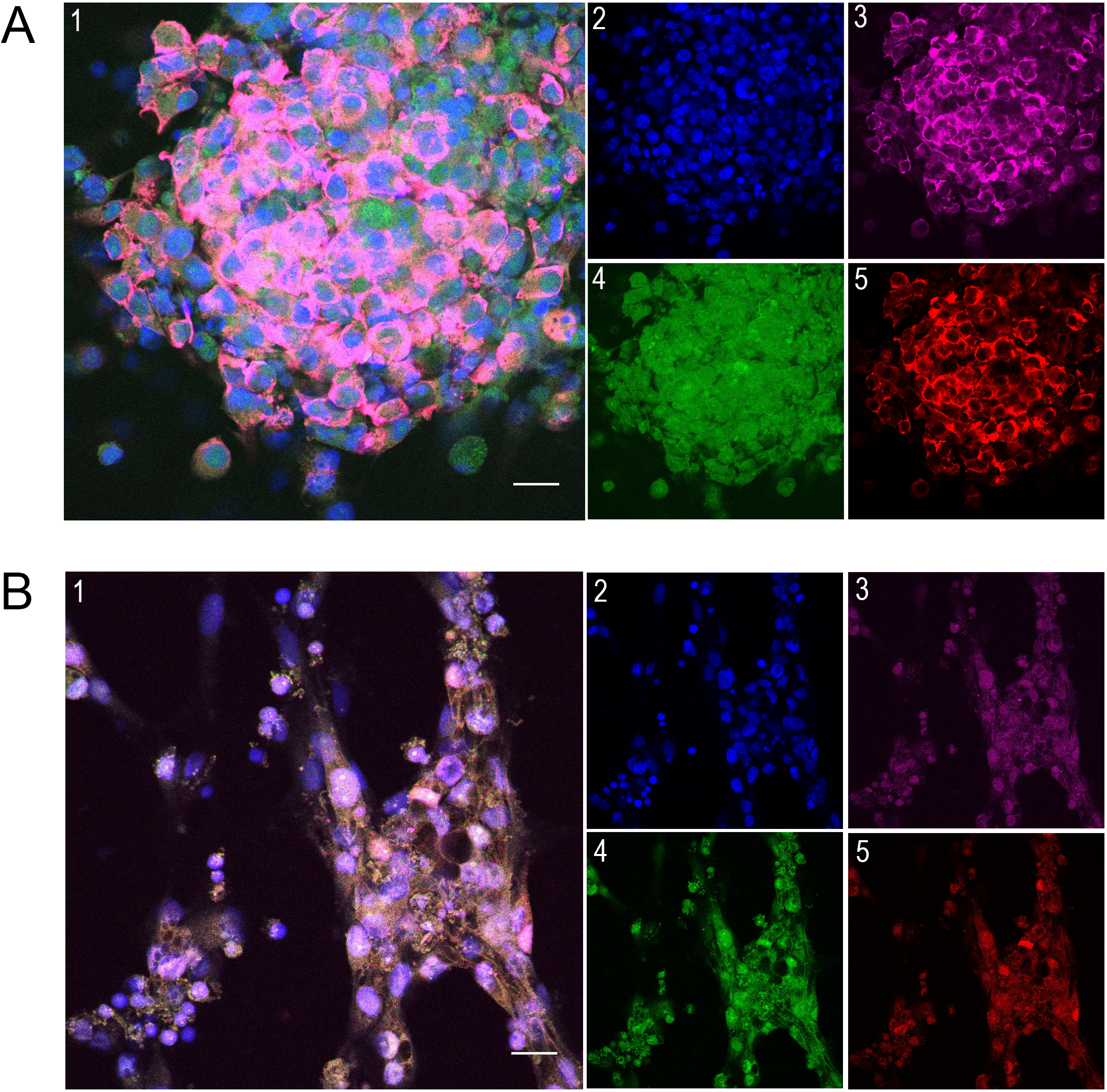


**Supplementary Figure 2: KEGG pathways**

*A,* Commonly enriched KEGG-pathways in RNA-Seq and CAGE data. KEGG pathways were sorted into thematically matching groups. Most pathways cluster together under cell fate, cancer pathways and signaling pathways. *B,* dnE47 induction related significantly altered KEGG pathways in RNA-Seq and CAGE data. Data showing commonly enriched pathways at 24h (group I), 48h and 72h (group II) and at 24h-72h (group III) after Doxycycline mediated dnE47-induction.


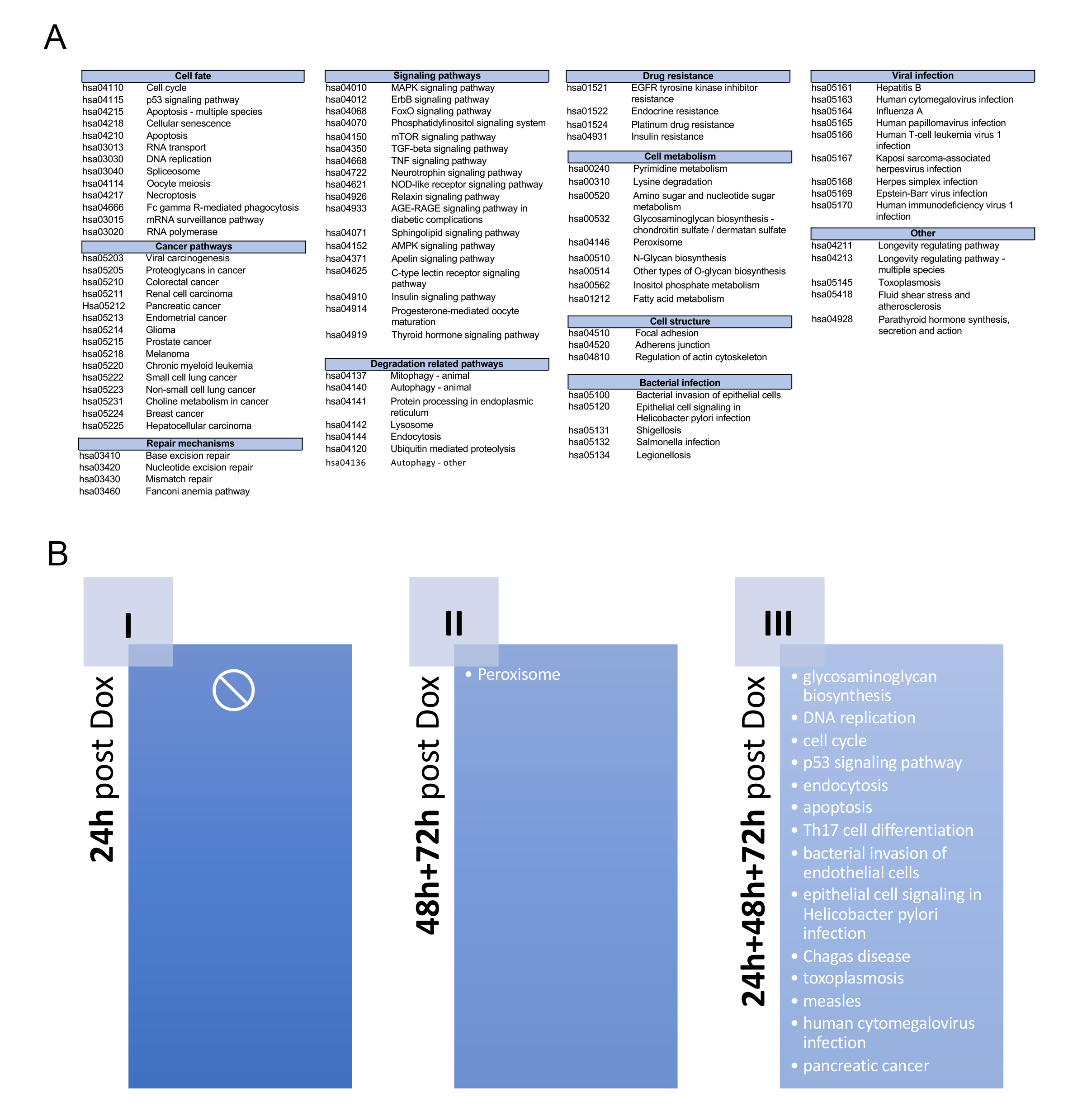


**Supplementary Figure 3: Increased replicative stress in bHLH expressing glioma**

Immunoblot for pRPA2 and RPA2 (LNZ308 RFP-dnE47 resp. RFP-control cells) showing reduced time-dependent expression of pRPA2 in doxycycline activated dnE47 samples, indicating higher replicative stress in bHLH expressing cells.


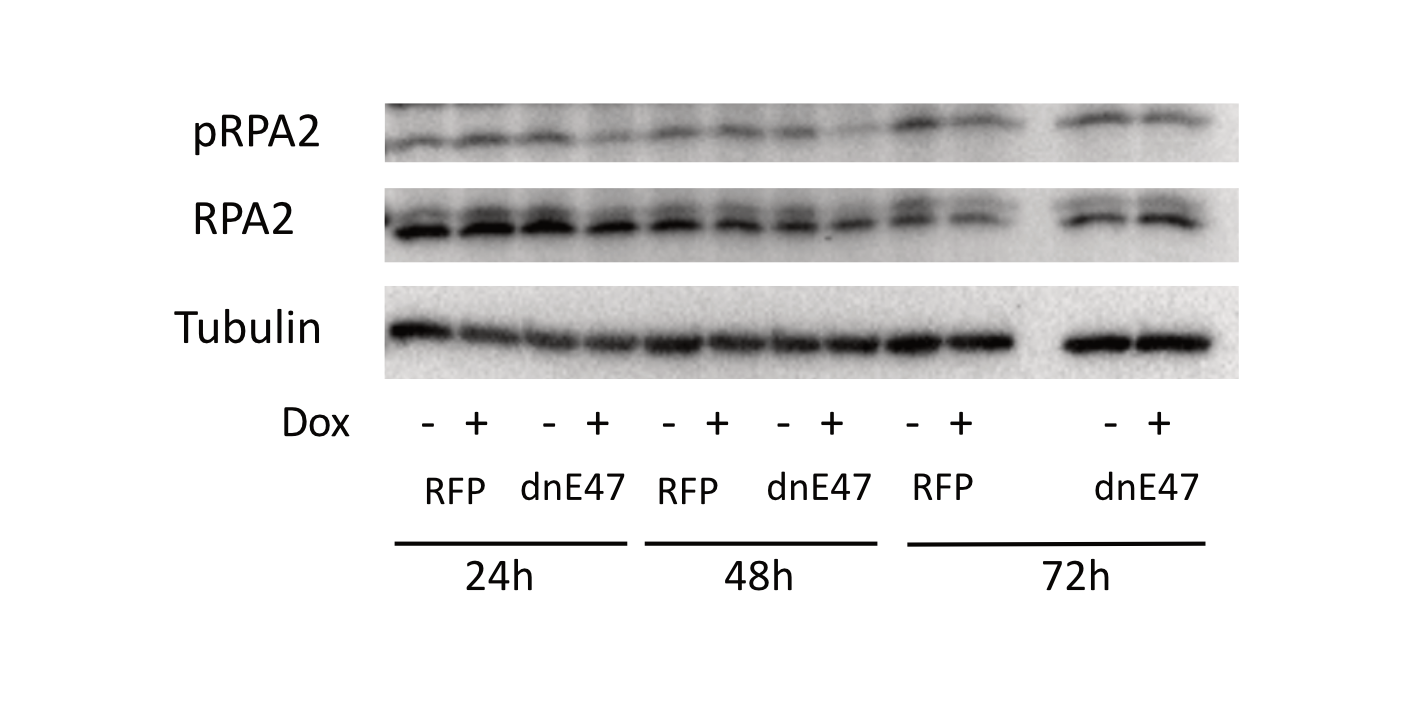

Supplement: vdaa115_suppl_Supplementary_Figures [file vdaa115_suppl_supplementary_figures.docx]
